# Metastases to the Male Breast: A Systematic Review

**DOI:** 10.3390/cancers18152440

**Published:** 2026-07-29

**Authors:** Nikolaos S. Georgopoulos, Maria Papadoliopoulou, Alexios Kozonis, Nikolaos V. Michalopoulos, Nikolaos Arkadopoulos

**Affiliations:** 1 4th Department of Surgery, Attikon University Hospital, Medical School, National and Kapodistrian University of Athens, 12462 Athens, Greece; 2 3rd Department of Obstetrics and Gynaecology, Attikon University Hospital, Medical School, National and Kapodistrian University of Athens, 1 Rimini Street, Chaidari, 12462 Athens, Greece; 3 First Propaedeutic Department of Surgery, Hippocration General Hospital, Medical School, National and Kapodistrian University of Athens, 12462 Athens, Greece

**Keywords:** breast metastases, male breast, extramammary malignancies, secondary breast tumors, immunohistochemistry, literature review

## Abstract

**Background/Objectives:** Breast metastases originating from extramammary malignant tumors represent an exceptionally rare clinical entity, particularly in the male population. This study aims to review relevant literature on secondary breast involvement in male patients, focusing on clinical presentation, imaging features, histological and immunohistochemical characteristics and management strategies. To date, no published systematic review focusing on this topic exists in the international literature. **Methods:** A systematic review of the available literature was conducted through a comprehensive search of the PubMed/MEDLINE database up to December 2025. A total of 376 records were identified, of which 98 articles met the inclusion criteria and were included in the analysis. **Results:** A total of 192 cases were identified, mainly from case reports and small case series. The mean age was 58 years, with similar distribution between breasts and occasional bilateral involvement. Breast metastases often occurred months to years after the primary tumor (mean 32 months) and typically indicated advanced disease with additional metastases. The most common primary tumors were prostate, lung, and melanoma. Diagnosis relied on biopsy with histological and immunohistochemical evaluation. Management was primarily directed at the underlying systemic disease, while surgery, although frequently performed, contributed mainly to diagnostic confirmation and local disease control. **Conclusions:** Histological identification and immunohistochemical characterization are crucial in the differential diagnosis between primary breast carcinoma and metastatic disease to the breast. The diagnosis of a metastatic lesion, rather than a primary breast malignancy, alters the therapeutic approach.

## Simple Summary

Breast metastases from extramammary malignancies in men are exceptionally rare and poorly characterized. As no previous systematic review has specifically addressed this topic, this study was conducted to synthesize the available evidence and improve understanding of its clinical, radiological, pathological, and therapeutic aspects. A systematic review of 98 studies identified 192 reported cases. The mean age at diagnosis was 58 years, and breast metastases occurred a mean of 32 months after the primary malignancy, often in the setting of disseminated disease. The most common primary tumors were prostate cancer, lung cancer, and melanoma. Diagnosis relied on histopathological examination and immunohistochemical profiling to distinguish metastatic lesions from primary breast carcinoma. Management was primarily directed at the underlying systemic malignancy, while surgery mainly served diagnostic and local control purposes. These findings provide a comprehensive reference for clinicians, support accurate diagnosis and treatment selection, and help address an important gap in the literature.

## 1. Introduction

Male breast cancer is a rare malignancy, accounting for approximately 1% of all breast cancers and 0.5–1% of all cancers in men. The mean age at diagnosis ranges between 60 and 70 years [1]. The most important risk factors for male breast cancer include genetic predisposition—particularly BRCA2 and, less frequently, BRCA1 mutations—conditions associated with hyperestrogenism such as obesity and chronic liver disease, endocrine disorders including hypogonadism, prior thoracic irradiation, and a family history of breast cancer. Clinically, the disease presents as a painless, firm mass, most often located in the retroareolar region and less frequently in the upper outer quadrant, where most of the glandular and connective tissue is concentrated. Additional clinical manifestations may include nipple retraction or inversion, skin changes such as infiltration or ulceration, nipple discharge, and axillary lymphadenopathy [2,3].

### Breast Metastases from Extramammary Malignancies in Men

Metastases to the breast from extramammary malignancies are rare. In the male population, the presence of a breast mass is most commonly attributable to gynecomastia or primary breast cancer, whereas metastatic lesions are even less frequent and often represent a diagnostic challenge [4]. Their rarity is attributed to the high proportion of fibrous tissue within the male breast and its relatively limited vascular supply, which make the tissue less favorable for the implantation of metastatic tumor cells [5].

The most common primary malignancies metastasizing to the male breast include lung cancer, prostate cancer, melanoma and lymphomas. Although prostate cancer was previously considered a relatively frequent source of breast metastases, it is now regarded as a rare cause, for reasons that will be discussed below.

Diagnosis requires a high index of clinical suspicion, as both the clinical presentation and imaging findings may closely resemble those of primary breast carcinoma. Recognition of the metastatic nature of the lesion is crucial, as it guides appropriate management by prioritizing systemic therapy for the primary malignancy and avoiding unnecessary breast surgery.

While more common in women than in men, breast metastases from extramammary malignancies remain rare, with reported frequencies ranging from 0.11% to 6.3% in histopathological series and 0.12% to 4.92% in imaging studies [6,7,8,9]. They can occur across a wide age spectrum and often mimic primary breast cancer, leading to potential misdiagnosis and inappropriate management [10].

The most common primary tumors metastasizing to the breast are cutaneous malignant melanoma and ovarian carcinoma, followed by less frequent sources such as gastric cancer, renal cell carcinoma, sarcomas, bronchogenic carcinoma, and laryngeal cancer [6].

Clinically, patients typically present with a solitary, rapidly enlarging, painless mass, often superficially located and without nipple retraction or discharge. Lesions are most frequently found in the upper outer quadrant and may be bilateral in up to 25% of cases.

On imaging, findings are non-specific but metastases tend to appear as well-circumscribed masses without spiculations or architectural distortion. On Magnetic Resonance Imaging (MRI), they often show T2 hyperintensity and rapid contrast enhancement, sometimes with washout kinetics, typically with more homogeneous features than primary breast cancers [4,11].

Breast metastases should be considered in the differential diagnosis of a breast mass, particularly in patients with a known extramammary malignancy. Histological confirmation with biopsy and immunohistochemistry is essential. Their presence usually indicates advanced systemic disease and is associated with poor prognosis [9].

Histopathological examination remains the cornerstone for the diagnosis of breast metastases. Macroscopically, metastatic lesions often appear as well-circumscribed nodules without infiltration of the surrounding breast tissue. Microscopically, the morphology of these lesions typically reflects the histological characteristics of the primary tumor and usually lacks features of in situ breast carcinoma, a finding that is particularly important in the differential diagnosis [12,13].

A combination of morphological evaluation and immunohistochemical analysis is essential for establishing the final diagnosis. The use of a panel of immunohistochemical markers reduces the risk of false-positive or false-negative results and facilitates the definitive distinction between primary breast carcinoma and metastatic disease.

The aim of the present study is to review the literature on reported cases of secondary breast involvement in male patients. Specifically, we seek to analyze the clinical presentation, imaging characteristics, and therapeutic approaches associated with metastatic breast lesions in men. A comprehensive understanding of these features is essential for accurate diagnosis and optimal patient management, given that metastatic lesions may closely mimic primary breast tumors. This review synthesizes the available demographic, clinical, diagnostic, and therapeutic data from published cases to provide a structured overview of this rare clinical entity.

## 2. Materials and Methods

A systematic literature search was conducted using the PubMed/MEDLINE database to identify studies reporting breast metastases in male patients originating from extramammary primary tumors, published up to December 2025. The search was performed using combinations of keywords and MeSH terms, including “extramammary breast metastases”, “male breast metastasis”, “breast metastasis in men” and “secondary breast tumors/neoplasms”.

Articles were included without time restrictions. Reports exclusively involving female patients or primary breast neoplasms were excluded. In addition, the reference lists of the selected articles were manually screened in order to identify additional relevant publications.

**Figure 1:**
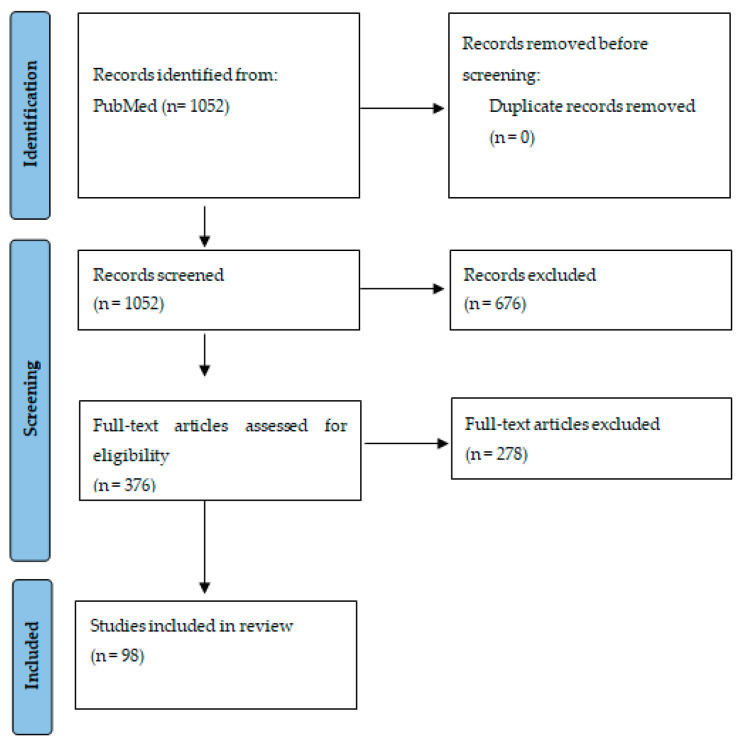
PRISMA 2020 flow diagram for systematic reviews.

The present systematic review was performed according to the Preferred Reporting Items for Systematic Reviews and Meta-Analyses (PRISMA) 2020 guidelines. A comprehensive and structured approach was employed for literature searching, study selection, data extraction, and evidence synthesis, ensuring consistency, transparency, and reproducibility throughout the review process. The PRISMA flow diagram (Figure 1) was used to document the identification, screening, eligibility assessment, and inclusion of studies. The completed PRISMA 2020 checklist is provided as Supplementary Material (Section S1, Tables S1 and S2). Risk of bias in the included studies was assessed using the Joanna Briggs Institute (JBI) critical appraisal tools appropriate for each study design (Section S2, Table S3). Each study was independently evaluated by two reviewers. No automation tools were used in the assessment process.

The initial search yielded 1052 results. After screening titles and abstracts and applying the predefined inclusion and exclusion criteria, 98 articles were considered relevant and were included in the final analysis.

The systematic review protocol was not prospectively registered in a public registry (e.g., PROSPERO). A review protocol was developed by the authors but was not publicly registered.

## 3. Results

Breast metastases in male patients represent a rare clinical and pathological entity. The international literature currently reports a total of 192 documented cases. Most publications consist of case reports and small case series, with the number of patients per study ranging from 1 to 17, while the majority of reports describe single cases.

### 3.1. Demographic and Clinical Characteristics of Patients

Analysis of the available demographic data showed that the mean age of men with metastatic disease to the breast was 58 years (range: 24–82 years), indicating that such metastases occur predominantly in middle-aged and older men.

The anatomical distribution of breast lesions was relatively balanced in cases where the information was available (n = 92), with 37 cases (40.2%) involving the right breast, 37 (40.2%) the left breast, and 18 (19.6%) presenting with bilateral involvement.

The temporal relationship between the diagnosis of the primary tumor and the appearance of the metastatic breast lesion demonstrated considerable variability. In some cases, the breast lesion appeared synchronously with the diagnosis of the primary tumor (5 cases), whereas in others occurred with a delay of up to 11 years after the initial diagnosis. The mean interval between primary tumor diagnosis and the detection of breast metastasis was 32.08 months, with a median of 24 months. The presence of additional metastases at the time of breast metastasis diagnosis is common, suggesting advanced-stage disease.

In at least 24 cases (12.5%), metastases to other organs were present at the time of diagnosis of the breast lesion, indicating advanced systemic disease.

The most frequent anatomical locations of metastatic lesions were the retroareolar region and the upper outer quadrant, possibly reflecting the relatively increased vascular density of the subcutaneous tissue in these areas.

Clinically, metastatic breast nodules tend to grow rapidly in size, and axillary lymph node involvement appears to be less frequent compared with primary breast carcinomas.

Among the 192 reported cases published in a total of 98 articles, 63 were single case reports and 35 were case series, highlighting the rarity of this condition and the challenges in collecting large datasets.

Comparison of cases reported before and after 2000 showed that prostate cancer was the most frequently reported primary tumor before 2000, whereas lung cancer became the predominant primary tumor after 2000.

### 3.2. Diagnostic Approach

The diagnosis of breast metastases was based on the combination of imaging findings and histological examination. Among the cases where biopsy information was available (n = 78), core needle biopsy was performed in 36 cases (46.2%), whereas fine needle aspiration (FNA) was used in 42 cases (53.8%).

### 3.3. Management

Surgical intervention was reported in 33 of the 46 cases in which therapeutic management was described, mainly for local control of the disease or for confirmation of the diagnosis. Systemic therapy, including chemotherapy, immunotherapy, or targeted therapy, was administered in 13 of the 46 cases, particularly in patients with additional metastatic sites outside the breast.

In at least 14 cases, the breast lesion was initially considered to be a primary tumor, emphasizing the importance of accurate histological and immunohistochemical evaluation in order to avoid inappropriate therapeutic management.

The most frequent primary tumors metastasizing to the male breast are prostate cancer (27.6%), lung cancer (19.8%), and melanoma (9.4%), which together account for approximately 57% of all reported cases. The distribution of primary tumors metastasizing to the breast in male patients is summarized in Table 1.

A summary of the demographic and clinical characteristics of the reported cases, including patient age, interval between primary tumor diagnosis and breast metastasis, laterality of breast involvement, biopsy methods, and treatment approaches, is presented in Table 2. Due to the retrospective nature of the available literature and incomplete reporting in several case reports, many clinical variables were available only for a subset of patients.

## 4. Discussion

### 4.1. Breast Metastases from Prostate Cancer

Metastasis to the breast from prostate cancer is extremely rare, typically occurring in patients with widely metastatic disease and/or a history of specific therapies. Clinically, patients present with a palpable mass, which may be unilateral or bilateral. Diagnosis is challenging due to frequent coexisting gynecomastia in patients receiving hormonal therapy. Mammographically, metastases appear as well-circumscribed, round or oval masses without characteristic microcalcifications. Architectural distortion is uncommon. Ultrasound typically shows hypoechoic solid masses, sometimes with increased vascularity on Doppler. MRI does not provide pathognomonic features to reliably distinguish metastatic from primary breast lesions. A definitive diagnosis requires histopathological confirmation through core needle biopsy, with immunohistochemical analysis playing a crucial role in accurate characterization. Management is primarily systemic, tailored to disease stage and tumor biology. Surgical excision of the breast lesion, though reported in many cases, does not improve survival and is reserved for diagnostic purposes or local complications.

Historically, estrogen therapy was used for metastatic prostate cancer (1940–1970), suppressing androgen production via hypothalamic–pituitary–gonadal axis inhibition. It has been proposed that prolonged high-dose estrogen exposure may induce hormonal changes in the male breast, potentially creating a microenvironment that facilitates metastatic implantation. Gynecomastia, vascular proliferation, and stromal hypertrophy facilitate seeding of prostate cancer cells. Breast involvement usually coexists with widespread metastases, indicating advanced-stage disease, with historically poor prognosis (median survival ~4 months). Modern combined androgen blockade and targeted systemic therapies have improved outcomes in selected patients.

A total of 53 cases of breast metastases originating from prostate cancer have been reported, representing the largest proportion of documented cases of breast metastases in men [5,12,14,15,16,17,18,19,20,21,22,23,24,25,26,27,28,29,30,31,32,33,34,35,36,37,38,39,40,41]. The clinicopathological characteristics of the reported cases of breast metastasis from prostate cancer are summarized in Table 3. Notably, the majority of these cases were reported before the 1990s, with 41 cases published up to 1991, corresponding to the era when high-dose estrogen therapy was commonly used for advanced prostate cancer. This temporal distribution is consistent with the previously described hypothesis that prolonged estrogen exposure may induce hormonal changes in the male breast, potentially creating a microenvironment more conducive to metastatic implantation. The earliest reports date back to 1957 by Benson, describing cases occurring in the context of estrogen therapy [34]. In some instances, metastatic lesions have been reported to mimic inflammatory breast cancer, as described by Njiaju et al. (2010), leading initially to diagnostic confusion [16].

A recent case by Zhang et al. (2025) [5] describes a 68-year-old man with long-standing metastatic prostate adenocarcinoma who presented with bilateral breast masses. Imaging (BI-RADS 4C) was suspicious, and core biopsy confirmed metastatic disease, with an immunoprofile of GATA-3 negative and ERG, prostate-specific acid phosphatase (PSAP), and NKX3.1 positive. The patient underwent bilateral tumorectomy and continued systemic therapy, remaining clinically stable. The authors note that prolonged hormonal treatment likely induced gynecomastia and a breast microenvironment favorable for metastatic implantation. Prostate-Specific Antigen (PSA) and PSAP positivity confirmed prostatic origin, while absence of markers such as ER, GCDFP-15, and CK7 helped exclude primary breast carcinoma [5].

PSA and prostatic acid phosphatase (PSAP) are highly sensitive markers for prostate carcinoma, being expressed in nearly 100% of tumors. Recent studies indicate that male breast cancers may express PSA in approximately 15% of cases, but they do not express prostate-specific acid phosphatase (PSAP) [42]. Estrogen receptors (ER), as well as markers such as GCDFP-15 and cytokeratin 7 (CK7), are rarely expressed in prostate carcinoma; therefore, their expression generally favors a diagnosis of primary breast carcinoma. The classical markers PSA and PSAP may be negative in poorly differentiated tumors or following androgen-deprivation therapy. The newer marker NKX3.1 demonstrates high sensitivity and specificity for prostatic origin, and its use in combination with PSA and PSAP increases diagnostic accuracy, particularly in challenging cases [43]. In addition, the markers P501S and PSMA may be useful in complex diagnostic scenarios [44,45]. It should also be noted that primary breast carcinomas in men may express PSA in up to 23% of cases [17,19].

### 4.2. Breast Metastases from Lung Cancer

A total of 38 cases of lung cancer metastases to the male breast have been reported [12,15,46,47,48,49,50,51,52,53,54,55,56,57,58,59,60,61,62,63,64,65,66,67]. With the decline in prostate cancer breast metastases due to improved systemic therapies, lung cancer has emerged as the most common primary malignancy causing male breast metastases. The reported cases of breast metastasis from lung cancer are summarized in Table 4.

The breast is generally an uncommon site of metastasis. This has been attributed, although not conclusively, to its relatively low vascularity and a microenvironment that may be less favorable for lung cancer cell implantation. Most reported cases involve small-cell or adenocarcinoma, while squamous cell and large-cell carcinomas are less common. Metastasis may occur hematogenously or via lymphatic connections between supraclavicular and axillary nodes, with hematogenous spread considered more frequent.

Core needle biopsy is essential, and immunohistochemistry is crucial to distinguish primary from metastatic tumors. Lung cancer metastases are negative for ER, PR, HER2, and GATA3, while adenocarcinomas frequently express Thyroid Transcription Factor-1 (TTF-1) and Napsin A. However, TTF-1 positivity alone is not definitive, as 2–3% of primary breast cancers may show weak positivity; GATA3 negativity supports lung origin.

The possibility of metastasis should be considered particularly for small-cell carcinoma in the breast, as primary small-cell breast carcinoma is extremely rare. Features favoring primary breast origin include coexisting DCIS or hormone receptor positivity. Morphological overlap exists between poorly differentiated breast carcinoma and large-cell lung carcinoma; some large-cell lung adenocarcinomas express TTF-1, while ER or GCDFP-15 expression favors breast origin. Clinical history and comparison with prior histology are critical. Lung adenocarcinomas may show characteristic patterns, such as alveolar growth or columnar mucin-secreting cells. In cases of squamous cell carcinoma, the expression of p40 or CK5/6 may assist in establishing the diagnosis [68,69,70,71].

HER2 expression can occur in male breast tumors, lung adenocarcinomas, and androgen-resistant prostate cancers. Therefore, HER2 positivity in a male breast tumor should prompt evaluation for possible metastatic origin from lung or prostate cancer [61].

Treatment is systemic and tailored to the lung cancer subtype and stage. Surgical excision of the breast lesion is primarily diagnostic or palliative and does not improve prognosis. Chemotherapy, immunotherapy, or targeted therapy (e.g., EGFR, ALK, ROS1, PD-L1-directed) constitute the mainstay of treatment. Prognosis is poor, as breast involvement generally indicates advanced systemic disease.

### 4.3. Breast Metastases from Melanoma

Melanoma accounts for ~9.4% of male breast metastases, with 18 published cases [12,36,62,72,73,74,75,76,77]. Cases of breast metastasis originating from melanoma are summarized in Table 5. Most patients have a prior history of cutaneous melanoma, underscoring the need for high clinical suspicion. Metastatic spread may occur hematogenously or via regional lymphatics, with ipsilateral breast involvement more common, suggesting local lymphatic dissemination. Clinically, lesions typically present as painless, rapidly enlarging masses, without nipple retraction or skin changes. Occasionally, cutaneous pigmentation or subcutaneous nodules may be observed. The breast lesion can occasionally be the first sign of melanoma recurrence [78,79,80].

Mammography shows well-circumscribed, oval or round masses without calcifications. Ultrasound reveals hypoechoic, solid or mixed lesions with increased Doppler vascularity. MRI demonstrates strong contrast enhancement with rapid washout, while PET-CT is useful for detecting other metastatic sites and staging [73,81].

Malignant melanocytes display pleomorphism, nuclear hyperchromasia, and often cytoplasmic melanin. Amelanotic cases require immunohistochemistry for diagnosis. Melanoma cells exhibit phenotypic plasticity, transitioning between epithelial, mesenchymal, and intermediate states, facilitating immune evasion, therapeutic resistance, and metastatic colonization [74,82,83].

Characteristic markers include S-100 (95–100%), HMB-45 (75–90%), Melan-A/MART-1, SOX10, and MITF. Markers of primary breast carcinoma (ER, PR, HER2, GATA3, GCDFP-15) are generally negative, aiding differential diagnosis. The main differential includes primary triple-negative breast carcinoma and metastases from other malignancies (e.g., lung, prostate).

Treatment focuses on systemic disease control. Surgery may be considered for isolated or symptomatic lesions but does not improve survival. Immunotherapy with anti-PD-1 (nivolumab, pembrolizumab) and anti-CTLA-4 (ipilimumab) is the mainstay. BRAF V600E-mutated tumors may benefit from targeted BRAF/MEK inhibitors (dabrafenib, trametinib). Prognosis is poor, reflecting advanced systemic disease [84].

### 4.4. Extranodal Involvement of Lymphomas in the Male Breast

Unlike solid tumors, lymphomas do not metastasize in the classical sense; rather, they disseminate systemically via the lymphatic network and bloodstream even at early stages. Extranodal involvement is significantly more common in non-Hodgkin lymphomas, which may exhibit aggressive spread to other organs, particularly in high-grade B-cell subtypes such as Diffuse Large B-Cell Lymphoma (DLBCL). In contrast, Hodgkin lymphoma typically spreads from one lymph node group to the immediately adjacent group following lymphatic flow, with distant extranodal involvement being relatively uncommon [85,86].

Breast involvement is rare and predominantly occurs in men with non-Hodgkin lymphoma. It may present as either a primary breast lymphoma—a rare entity, usually DLBCL—or as secondary involvement in the context of systemic disease [87]. Lymphogenous metastases to the breast, which differ from hematogenous metastases, often present as diffusely and heterogeneously increased density within subcutaneous fat and glandular tissue. Secondary findings may include skin thickening, lymphedema, and regional lymph node enlargement, features that resemble inflammatory breast cancer [88,89]. Microcalcifications, commonly observed in inflammatory breast cancer, are rare in lymphovascular metastases [89,90].

Ultrasound imaging typically demonstrates diffuse skin thickening, loss of subcutaneous fat, and dilated lymphatic vessels, without a clearly defined primary mass. These findings result from edema caused by mechanical obstruction of lymphatics by the tumor. High-grade lymphomas tend to display a more diffuse, infiltrative parenchymal pattern [91].

Lymphoma accounts for approximately 3.1% of metastatic tumors in the male breast, with only six cases documented in the international literature (Table 6) [62,88,92,93], making it the fourth most common metastatic malignancy in this population, after prostate, lung cancer, and melanoma, with a comparatively lower mean age at presentation.

### 4.5. Gastrointestinal Malignancies

Metastases to the breast from gastrointestinal malignancies, including gastric, colon, and rectal cancers, have been reported primarily as isolated case reports or small series, reflecting both their rarity and the diagnostic challenges associated with their detection [6,15,94,95,96,97,98,99,100,101,102,103,104]. Clinically, these lesions often present as rapidly growing, painless, subcutaneous or superficial nodules that lack the classic imaging features of primary breast carcinoma, such as microcalcifications or skin changes, complicating diagnosis in male patients.

Immunohistochemistry is critical for confirming metastatic origin, as histological morphology alone is rarely sufficient to distinguish these lesions from primary breast tumors. Metastases from gastric carcinoma typically exhibit a CK7-negative or focally positive, CK20-positive profile with strong, diffuse CDX2 expression, reflecting intestinal-type differentiation. Depending on histologic subtype, gastric mucins such as MUC1, MUC5AC, and MUC6 may be expressed, while HER2 expression is possible but does not aid in differential diagnosis [105].

Metastases from colorectal carcinomas display a more characteristic immunophenotype, with CK7 usually negative, CK20 positive, strong CDX2 expression, and frequent SATB2 positivity, a highly specific marker of colorectal origin [106]. Markers typically associated with primary breast carcinoma, such as ER, PR, HER2, GATA3, and mammaglobin, are generally negative in gastrointestinal metastases. The absence of in situ components, such as DCIS, further supports a metastatic rather than primary breast origin. The clinicopathological characteristics of the reported cases of breast metastasis from gastrointestinal malignancies in men are summarized in Table 7.

### 4.6. Urinary Tract Malignancies

Metastases to the breast from urinary tract malignancies, including renal cell carcinoma, ureteral carcinoma, and urothelial carcinoma of the bladder, represent an exceptionally rare entity (Table 8) [36,67,107,108,109,110,111,112,113]. Immunohistochemistry is the cornerstone for identifying the primary site. Renal cell carcinoma, particularly the clear cell subtype, is characterized by strong expression of PAX8, vimentin, and often CD10, while typically negative for CK7, CK20, GATA3, ER, PR, HER2, and mammaglobin [107].

In contrast, metastases from ureteral carcinoma and, more commonly, bladder urothelial carcinoma exhibit a characteristic urothelial immunoprofile, with GATA3, CK7, CK20, p63, p40, and uroplakin II/III representing the most reliable markers [114,115]. Although GATA3 is also expressed in some primary breast tumors, concurrent positivity for CK20, uroplakin, and p63—which are absent in most breast carcinomas—supports urothelial origin. CK5/6 is frequently expressed in urothelial metastases but may also be observed in triple-negative primary breast cancers, particularly basal-like subtypes [116]. Therefore, CK5/6 is not a specific marker, and differentiation between urothelial metastasis and triple-negative breast carcinoma requires a combined immunohistochemical panel, including markers such as GATA3, p63, and uroplakin II. Additionally, negativity for ER, PR, and HER2, as well as absence of DCIS, further supports metastatic deposition rather than a primary breast malignancy.

The remaining rare cases and isolated case reports of breast metastases in male patients are summarized in Table 9.

### 4.7. Immunohistochemical Markers in the Differential Diagnosis

Table 10 summarizes the main immunohistochemical (IHC) markers used to distinguish breast metastases in male patients according to the primary tumor. The use of this IHC panel allows reliable differentiation between metastatic lesions and primary breast cancer, as well as assessment of the origin from other malignancies. Systematic application of these markers significantly aids accurate pathological evaluation and guides appropriate oncologic management.

**Figure 2:**
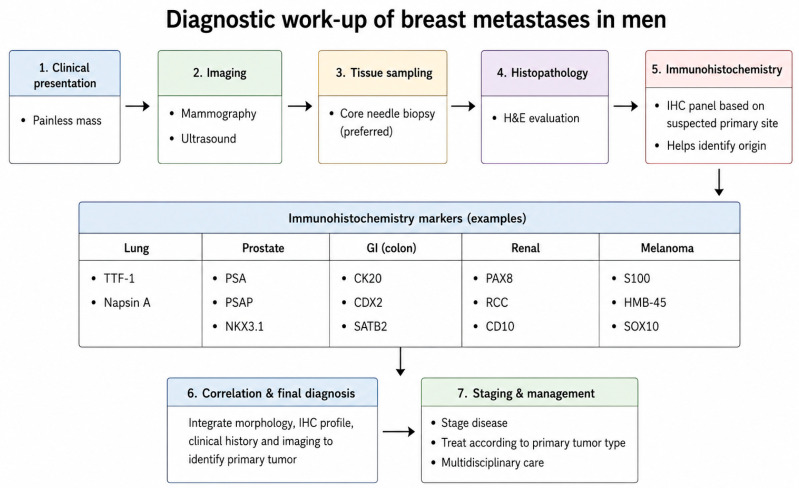
Diagnostic work-up of breast metastases in men.

Figure 2 illustrates a practical diagnostic algorithm for the evaluation of suspected breast metastases in men, emphasizing the central role of immunohistochemistry in determining the primary tumor origin.

### 4.8. Limitations

This study has several limitations. Most available data derive from case reports and small case series, precluding robust statistical analysis and limiting the generalizability of the findings. Case reports are also subject to incomplete reporting, with variable availability of clinical information, imaging findings, immunohistochemical profiles, treatment details, and follow-up outcomes. Significant heterogeneity exists regarding clinical presentation, diagnostic approaches, including biopsy techniques, pathological assessment, and immunohistochemical panels, which may limit direct comparisons between reported cases. Furthermore, the rarity of this condition and the absence of population-based studies prevent accurate estimation of its incidence. The substantial heterogeneity among the included studies and the limited number of comparable cases precluded formal meta-analysis. Additionally, publication bias is likely, as rare or complex cases are more likely to be reported.

## 5. Conclusions

Breast metastases in male patients from non-breast primaries are extremely rare but clinically important, with most evidence limited to case reports and small series. The most common primary sites are prostate cancer, followed by lung cancer, melanoma, lymphoma, and gastrointestinal tumors.

They usually indicate advanced systemic disease and carry a poor prognosis. Diagnosis relies on biopsy and immunohistochemistry to distinguish metastases from primary breast cancer using tumor-specific markers (e.g., PSA, PSAP, NKX3.1 for prostate; TTF-1, Napsin A for lung; Melan-A for melanoma).

Accurate differentiation is essential, as management is mainly systemic and guided by the primary tumor, while surgical intervention has a limited role, primarily for diagnostic confirmation or local disease control and does not improve overall survival. A multidisciplinary approach is crucial, and further multicenter studies are needed to better understand metastatic behavior according to primary tumor type, ultimately enhancing therapeutic decision-making.

## Figures and Tables

**Table 1:** Distribution of primary tumors metastasizing to the breast in male patients (n = 192).

Primary Tumor Site	Number of Cases (n)	Percentage (%)
Prostate	53	27.6%
Lung	38	19.8%
Melanoma	18	9.4%
Lymphoma	6	3.1%
Stomach	5	2.6%
Colon	5	2.6%
Rectum	4	2.1%
Kidney	4	2.1%
Urothelial carcinoma	3	1.6%
Multiple myeloma	3	1.6%
Pharynx	3	1.6%
Esophagus	2	1%
Ureter	2	1%
Hepatocellular carcinoma	2	1%
Skin (non-melanoma)	1	0.5%
Lymphoblastic leukemia	1	0.5%
Penis	1	0.5%
Testis	1	0.5%
Larynx	1	0.5%
Salivary gland	1	0.5%
Oral cavity	1	0.5%
Pancreas	1	0.5%
Glioma	1	0.5%
Synovial sarcoma	1	0.5%
Cases in which the primary tumor was not reported	34	17.7%

**Table 2:** Summary of demographic and clinical characteristics of reported cases of breast metastases in male patients.

Parameter	Summary of Reported Data
Number of reported patients	192
Number of included articles	98
Mean age at diagnosis of breast metastasis	58 years (range: 24–82 years)
Interval between primary tumor diagnosis and breast metastasis	Mean: 32.08 months; Median: 24 months (range: synchronous presentation to 11 years)
Laterality of breast involvement (n = 92)	Right breast: 37 (40.2%) Left breast: 37 (40.2%) Bilateral involvement: 18 (19.6%)
Presence of additional metastases at diagnosis	≥24 cases (12.5%)
Biopsy method (n = 78)	Core needle biopsy: 36 (46.2%) Fine needle aspiration: 42 (53.8%)
Initial misdiagnosis as primary breast tumor	14 cases
Treatment reported (n = 46)	Surgical treatment: 33 (71.7%) Systemic therapy: 13 (28.3%)

**Table 3:** Reported Cases of Male Breast Metastases from Prostate Cancer.

No	Authors	Year	No. of Cases	Age (yrs)	Site	Diagnosis	Management/Outcome
1	Zhang et al. [5]	2025	1	68	Bilateral, 7 years after initial diagnosis	Prostate adenocarcinoma with bone metastases, on endocrine therapy. GATA3 (−), ERG (+), PSAP (+), NKX3.1 (+). Genetic testing: BRCA2 (+), AR (+)	Bilateral lumpectomy, chemotherapy, anti-androgen therapy
2	Mokoala et al. [14]	2019	1	80	Bilateral, detected on PET-CT	Castration-resistant prostate cancer, core biopsy	metastatic disease
3	Shah et al. [15]	2014	2	60–69	–	–	–
4	Njiaju et al. [16]	2010	1	78	Right breast, initially considered inflammatory breast cancer	Prostate adenocarcinoma with bone metastases. Core biopsy (non-diagnostic) and skin biopsy (diagnostic). IHC confirmation (PSA, AMACR) after 3 months due to non-response to initial therapy	Hormonal therapy—androgen blockade
5	Monaco et al. [17]	2009	1	60	Right breast	FNA initially, excisional biopsy, IHC confirmation. PSA, P504S, AR (−). GCDFP-15, mammaglobin, ER, PR (−)	–
6	Fulciniti et al. [18]	2007	1	70	Right breast, 3 years after initial diagnosis	FNA, IHC confirmation (PSA expression)	–
7	Lee et al. [12]	2007	1	75	–	Metastasis 5.6 years after initial diagnosis	–
8	Cheng et al. [19]	2006	1	79	Bilateral, right breast 6 cm, 2 years 8 months after initial diagnosis	Core biopsy, IHC confirmation (PSA, PSAP)	–
9	Hsieh et al. [20]	2005	1	71	Right breast, 2.5 cm	Excisional biopsy, IHC confirmation (PSA)	–
10	Sahoo et al. [21]	2001	1	78	Right breast, 5 cm, 12 months after initial diagnosis	FNA non-diagnostic, simultaneous papillary carcinoma of the breast and metastatic prostate adenocarcinoma (distinction using GCDFP-15, ER, PR, CK7)	Modified radical mastectomy
11	Yan et al. [22]	2000	1	81	Bilateral, multiple	FNA (PSA, PSAP positive; ER, PR negative)	Metastatic liver and bone disease
12	Ramamurthy et al. [23]	1991	1	64	Bilateral, multiple and gynecomastia	FNA	Under estrogen therapy
13	Moldwin et al. [24]	1989	1	45	Mass (side not specified) and bilateral gynecomastia	Core biopsy	Under estrogen therapy
14	Toombs et al. [25]	1977	9	Mean 63.1	–	–	8/9 under estrogen therapy
15	Green et al. [26]	1991	2	63/61	Right breast 2.5 cm/Bilateral	Under estrogen therapy. Excisional biopsy/core biopsy	Excisional biopsy/bilateral lumpectomies
16	Salyer et al. [27]	1973	11	–	–	–	metastatic disease
17	Allen et al. [28]	1991	1	–	–	IHC	metastatic disease
18	Drelichman et al. [29]	1980	3	71/71/75	Bilateral	–	metastatic disease, Under estrogen therapy
19	Naritoku et al. [30]	1983	2	78/68	Right/Left	IHC	Under estrogen therapy 1. Simple mastectomy 2. Excisional biopsy
20	Kitano et al. [31]	1983	1	59	Right breast, 4 months after initial diagnosis		Under estrogen therapy Lumpectomy
21	Kumar et al. [32]	1986	1	78	Right breast, 3 months after initial diagnosis	Biopsy, IHC	under estrogen therapy
22	Hesdorffer et al. [33]	1987	1	–	–	IHC	–
23	Benson [34]	1957	1	–	Gynecomastia		Under estrogen therapy. Mastectomy and lymph node dissection
24	Campbell et al. [35]	1962	1	78	Bilateral	IHC, PAP and tissue acid-glycerophosphatase	Lumpectomy
25	Deeley [36]	1965	1	43	Right breast	–	–
26	Hartley [37]	1971	1	62	Bilateral	–	Under estrogen therapy
27	Lome et al. [38]	1970	1	64	Right breast, 4 cm, 33 months after initial diagnosis	IHC (PSAP)	Under estrogen therapy. Simple mastectomy
28	Pribe et al. [39]	1963	1	67	–	–	Under estrogen therapy. Simple mastectomy
29	Scott et al. [40]	1974	1	75	Bilateral, 2 years after initial diagnosis	–	Right mastectomy and excisional biopsy
30	Seipel et al. [41]	1972	1	65	Bilateral, 44 months after initial diagnosis in right breast, 60 months in left breast	–	Under estrogen therapy. Bilateral lumpectomies

**Table 4:** Reported Cases of Male Breast Metastases from Lung Cancer.

No.	Authors	Year	No. of Cases	Age (yrs)	Site	Diagnosis	Management/Outcome
1	Hashmi et al. [46]	2025	3	Median 72	–	lung adenocarcinoma	–
2	Lahham et al. [47]	2025	1	56	Right breast 1.4 cm, right axillary lymph nodes	Core biopsy: poorly differentiated lung adenocarcinoma, CK7, TTF1, Napsin-A, PD-L1, ROS1 (+); GATA3, ER, PR, HER2 (−)	Initial chemotherapy discontinued due to neutropenia; targeted therapy for EGFR mutation with good response
3	Koh et al. [48]	2024	1	51	Bilateral	Core biopsy: ALK-positive NSCLC, on chemotherapy	Bilateral lumpectomy, no recurrence at 8 months
4	Klingen et al. [49]	2009	1	70	Right breast 0.9 cm	Lung adenocarcinoma	Died 4 months after diagnosis of breast metastasis
5	Lin et al. [50]	2016	1	49	Right breast, 1.5 years after initial diagnosis	Core biopsy: poorly differentiated small cell lung carcinoma, arising from adenocarcinoma during EGFR-TKI therapy. Chromogranin A, synaptophysin, CD56, TTF-1 (+); ER, GCDFP-15, HER2 (−); breast lesion had same EGFR exon 21 mutation	Chemotherapy, bone and brain metastases, died 8 months later
6	Wan X et al. [51]	2020	1	81	Right breast 2.6 cm	Lung adenocarcinoma	–
7	Grigoropoulos et al. [52]	2019	1	57	Right breast, upper outer quadrant, 28 months after initial diagnosis	NSCLC	Modified radical mastectomy; poor prognosis
8	Erhamamci et al. [53]	2016	1	74	Right breast, 1.3 cm, retroareolar, synchronous diagnosis, incidental finding on PET	Excisional biopsy: lung adenocarcinoma	Chemotherapy, died 11 months later
9	Hachisuka et al. [54]	2014	1	60	Right breast, 4 cm, retroareolar, erythema, skin edema; breast MRI negative for primary malignancy, CT suggestive of lung primary	Core biopsy; initially diagnosed as primary breast carcinoma; lung biopsy confirmed. ER, PR, HER2 (−); IHC: GCDFP15 (−)	Breast metastasis responded to chemotherapy; died 4 months later
10	Shah et al. [15]	2014	3	60–69	–	Core biopsy	–
11	Altintoprak et al. [55]	2011	1	47	Right breast, synchronous	Small cell lung carcinoma; IHC: ER, PR, HER2, CK7, CK20 (−), chromogranin (+), synaptophysin (+), pancytokeratin (focal), TTF-1 (+)	Mastectomy, chemotherapy
12	Ucar et al. [56]	2007	1	–	–	NSCLC	–
13	Wood et al. [57]	2008	1	67	Right breast, lower outer quadrant, cervical lymphadenopathy	Lung adenocarcinoma; core biopsy: CK7 (+), TTF-1 (+); ER, PR (−)	–
14	Lee et al. [12]	2007	1	58	3 months from initial diagnosis to metastasis	Large cell lung carcinoma	–
15	Gómez-Caro et al. [58]	2006	1	65	Right breast, 18 months after right pneumonectomy	FNA and core biopsy: NSCLC, CK4 (+), CK7 (+), TTF-1 (−)	Mastectomy and lymph node dissection; adjuvant chemotherapy; disease-free 18 months; surgery chosen for local disease control
16	Hejmadi et al. [59]	2003	1	72	–	FNA: small cell lung carcinoma	–
17	Ramar et al. [60]	2001	1	56	Right breast, 4 years after initial diagnosis	Moderately differentiated lung adenocarcinoma; CH20, CK7, CAM 5.2, ER, PR, CDP (−)	2 cycles of chemotherapy and palliative care
18	David et al. [61]	2002	1	76	Right breast, subareolar, synchronous diagnosis; initially considered breast primary; staging CT found right lung nodule	FNA: poorly differentiated large cell lung carcinoma, HER2 2+, ER, PR (−); lung biopsy confirmed	Excisional biopsy
19	Siddiqui et al. [62]	2002	9	–	synchronous diagnosis in 2/9	FNA	–
20	Muttarak et al. [63]	1998	2	46/63	In one patient initially diagnosed as primary breast cancer	Lung carcinoma	Chemotherapy
21	Domanski [64]	1996	1	51	Right breast, 3 cm subareolar, gynecomastia, synchronous	Large cell lung carcinoma	–
22	Cooper et al. [65]	1994	1	65	Right breast, 2.5 cm	Core biopsy: large cell lung carcinoma	–
23	Verger et al. [66]	1992	1	63	Right breast, 4 cm, 18 months after initial diagnosis	FNA	Chemotherapy—radiotherapy
24	Sneige et al. [67]	1989	2	69/56	Right breast 3 cm, upper outer quadrant, 1 year after initial diagnosis/Left breast, skin retraction, synchronous	FNA; squamous cell carcinoma of lung/poorly differentiated lung carcinoma	Chemotherapy in the second patient, complete response at 4 months

**Table 5:** Reported Cases of Male Breast Metastases from Melanoma.

No	Authors	Year	No. of Cases	Age (yrs)	Site	Diagnosis	Management/Outcome
1	Santana Valenciano et al. [72]	2023	2	37/56	Cutaneous melanoma/Choroidal melanoma	–	Case 1: Mastectomy and lymph node dissection; survival 40 months; Case 2: no surgery; survival 132 months
2	Kang et al. [73]	2014	1	62	Right breast, upper outer quadrant, 4 cm; history of excised postauricular melanoma 8 years prior	FNA, IHC: HMB-45, Melan-A, S-100 (+)	Lumpectomy and SLNB
3	Bacchi et al. [74]	2013	3	53/57/69	Right breast/unknown in other two cases	Core biopsy (case 1); initial misdiagnosis	Mastectomy (cases 2–3)
4	Lee et al. [12]	2007	1	73	–	Melanoma (spindle cell type)	–
5	Garza-Guajardo et al. [75]	2005	1	26	5 cm, retroareolar; melanoma 3 years prior	FNA; Cytokeratin (−), vimentin, S-100, HMB45 (+)	Mastectomy
6	Shukla et al. [76]	2005	1	38	Right foot melanoma 2 years prior	FNA	–
7	Siddiqui et al. [62]	2002	4	–	–	FNA	
8	Cangiarella et al. [77]	1998	2	56/71	Right breast, lower outer quadrant/Left breast, lateral, history of left dorsal melanoma		
9	Deeley [36]	1965	3	60/55/42	Right breast/ Left breast/-		

**Table 6:** Reported Cases of Extranodal Involvement of Lymphomas in the Male Breast.

No.	Authors	Year	No. of Cases	Age (yrs)	Site	Diagnosis	Management/Outcome
1	Kalli et al. [92]	2015	1	52	Right breast, retroareolar, detected on follow-up PET/CT 19 months after initial diagnosis	Core biopsy: low-grade B-cell lymphoma	–
2	Mun et al. [88]	2014	1	34	Right breast, lower outer quadrant	Core biopsy: NK/T-cell lymphoma	–
3	Fraulini et al. [93]	2005	1	26	Right breast lesions, parasternal region, and bilateral axillary lymph nodes; detected on PET/CT follow-up 3 years after initial diagnosis	FNA: Hodgkin lymphoma	Chemotherapy and radiotherapy; no recurrence at 4 months
4	Siddiqui et al. [62]	2002	3	–	–	FNA: B-cell lymphoma	–

**Table 7:** Reported Cases of Male Breast Metastases from Gastrointestinal malignancies.

No.	Authors	Year	No. of Cases	Age (yrs)	Site	Diagnosis	Management/Outcome
1	Cheng et al. [94]	2020	1	57	Right breast, 3.9 cm, 6 months after initial diagnosis	Core biopsy: low-grade adenocarcinoma of lower rectum, CK7 (−), CK20 (+)	Chemotherapy, bone metastases; died 3 months later
2	Gur et al. [95]	2020	1	47	Bilateral, upper outer quadrants, 2 years after initial diagnosis	Core biopsy: mucinous adenocarcinoma of rectum; genetic testing: BRCA1 (+)	Bilateral mastectomy and chemotherapy; PET scan 6 months later showed axillary nodes positive on FNA
3	Wang T et al. [96]	2011	1	38	Right breast, 6.2 cm, previously operated rectal cancer 7 yrs ago	Core biopsy: initially considered primary breast carcinoma; surgical specimen compatible with mucinous rectal neoplasm, CK7 (–), GCDFP-15 (–), CK20 (+)	Neoadjuvant chemotherapy and mastectomy; after 3 cycles chemotherapy, liver lesions detected
4	Moreno-Astudillo et al. [6]	2019	1	63	Bilateral, retroareolar, nipple retraction, 1 month after initial diagnosis	Core biopsy: gastric adenocarcinoma with signet-ring cells	–
5	Kubo et al. [97]	2018	1	72	Right breast, retroareolar, 4 cm, 2 years after initial diagnosis	Core biopsy: poorly differentiated gastric adenocarcinoma, ER, PR, GCDFP-15 (–), CK7 (+), CK20 (+)	Mastectomy and chemotherapy; pelvic recurrence 15 months later
6	Tsung et al. [98]	2017	1	82	Right breast, 3.6 cm, 2.5 years after initial diagnosis; concurrent lung nodule	Core biopsy: moderately differentiated colorectal adenocarcinoma; initially considered primary breast tumor; ER, PR, HER2, CK7, CK20, GATA-3 (–), CDX-2 (+)	Simple mastectomy and oral capecitabine; refused lung biopsy; alive at 6 months
7	Shah et al. [15]	2014	7	60–69	–	Core biopsy: esophagus 2, stomach 2, pancreas 1, colon 1, rectum 1	–
8	Ho et al. [99]	2009	1	50	Right breast, 1.5 cm; prior right colectomy 6 yrs ago; recent completion of chemotherapy for lung and brain metastases	Core biopsy, Colon adenocarcinoma	Chemotherapy and immunotherapy; alive 1 year later
9	Bruscagnin et al. [100]	1997	1	66	Left breast, retroareolar, 2.2 cm, 2 years after initial diagnosis	Colon adenocarcinoma	–
10	Lear et al. [101]	1980	1	66	Left breast, 3 cm, 1 year after initial diagnosis	Colon adenocarcinoma	–
11	Lee et al. [102]	2011	1	71	Left breast	Hepatocellular carcinoma	–
12	Lo et al. [103]	2004	1	–	–	Hepatocellular carcinoma	Metastatic disease. Died 1 month after breast metastasis diagnosis
13	Hamby et al. [104]	1991	1	46	–	Undifferentiated gastric carcinoma	–

**Table 8:** Reported Cases of Male Breast Metastases from urinary tract cancers.

No.	Authors	Year	No. of Cases	Age (yrs)	Site	Diagnosis	Management/Outcome
1	Hoang et al. [107]	2025	1	55	Left breast; prior left nephrectomy 3 yrs ago	Core biopsy; initially considered primary breast tumor. Surgical specimen immunohistochemistry: CD10, Vimentin, CAIX, PAX8 (+), GATA3, CK20, CK7 (–). Clear cell renal carcinoma	Wide local excision; no adjuvant therapy; disease-free 3 years later
2	Yoon et al. [108]	2021	1	59	Bilateral painful breast nodules (2 cm R, 4 cm L), left breast erythema; left axillary nodes	Core biopsy: low-grade urothelial carcinoma of bladder with peritoneal and bone metastases	9 months post-chemotherapy
3	Ishikawa et al. [109]	2020	1	74	Right breast, 5.5 cm, retroareolar, 2.5 yrs after initial diagnosis	Core biopsy; initially considered primary breast. Ureteral carcinoma without intraductal lesion; final diagnosis after multiple pathology reviews	Mastectomy and SLNB; multiple lung metastases 2 months later
4	Roy et al. [110]	2019	1	82	Right breast, 3 yrs after initial diagnosis	Core biopsy: urothelial carcinoma of proximal ureter	Brain metastases
5	Sippo et al. [111]	2016	1	76	Right breast; PET/CT finding; prior cryoablation 7 yrs ago for right kidney carcinoma; undergoing chemotherapy for lung SCC	Core biopsy: renal cell carcinoma	–
6	Cappabianca et al. [112]	2000	1	69	Right breast, 2 cm, upper outer quadrant, nipple retraction	Urothelial carcinoma of bladder; histology negative within ducts; subcutaneous spread; immunohistochemistry confirmed diagnosis	Lumpectomy
7	Gibbons et al. [113]	1995	1	73	Right breast, upper outer quadrant; synchronous diagnosis	FNA; initially considered primary breast; renal cell carcinoma	Mastectomy and adjuvant chemotherapy
8	Sneige et al. [67]	1989	1	55	Right breast, 3 cm, retroareolar; 4 yrs after initial diagnosis	FNA: urothelial carcinoma	Simple mastectomy; disease-free 8 months later
9	Deeley [36]	1965	1	67	Right breast	Renal carcinoma	–

**Table 9:** Reported Cases of Male Breast Metastases from Rare Tumors or Cases Not Classified Elsewhere.

No.	Authors	Year	No. of Cases	Age (yrs)	Site	Diagnosis	Management/Outcome
1	Portaluri et al. [117]	2022	1	81	Right breast, upper outer quadrant, 3.5 cm; right axillary lymph nodes	Core biopsy: cutaneous squamous cell carcinoma	Wide local excision and lymph node dissection; no recurrence 1 yr later
2	Wan X et al. [51]	2020	1	61	Right breast, 6 mm, upper outer quadrant	Neuroendocrine tumor of larynx	–
3	Sun et al. [118]	2018	2	45/46	–	Undifferentiated carcinoma of nasopharynx	–
4	Khazai et al. [119]	2016	1	72	Right breast, 3 nodules; history of parotid tumor with bone and skin metastases	Parotid salivary gland carcinoma	Chemotherapy
5	Franceschini et al. [120]	2016	1	49	Right breast, 2 cm, periareolar; 3 yrs after initial diagnosis	FNA non-diagnostic; moderately differentiated penile SCC	Mastectomy and chemotherapy; pulmonary metastases; died 8 months later
6	Narese et al. [121]	2014	1	57	–	Core biopsy: unknown primary, large cell neuroendocrine carcinoma	–
7	Kohi et al. [122]	2013	1	36	Right breast, 1.5 cm, upper inner quadrant; 13 months after initial diagnosis	FNA: testicular leiomyosarcoma; IHC SMA, desmin	Mastectomy; widespread metastatic disease
8	Alacacioglu et al. [123]	2012	1	58	–	Anaplastic oligodendroglioma	–
9	López Guerra et al. [124]	2008	1	54	Right breast, 4 cm, upper inner quadrant; 6 months after initial diagnosis	Well-differentiated SCC of floor of mouth	Mastectomy and SLNB; no recurrence 33 months later
10	Prieto et al. [125]	2005	1	59	Right breast, retroareolar, 3 cm; 1 yr after initial diagnosis	Core biopsy SCC of hypopharynx	–
11	Nair et al. [126]	2005	1	24	Right breast, 2.5 cm; PET finding; history of soft tissue sarcoma R upper limb	Soft tissue sarcoma	–
12	Siddiqui et al. [62]	2002	1	–	–	FNA: multiple myeloma	–
13	Chim et al. [127]	2000	1	23	Right breast, 2 cm; 18 months after initial diagnosis; disease remission on maintenance therapy	Lymphoblastic leukemia	Chemotherapy and radiotherapy; CNS relapse; died 7 months later
14	Deshpande et al. [128]	1999	1	55	Subareolar, 4 cm	FNA: multiple myeloma	–
15	Domanski [64]	1996	1	82	Right breast, 2 cm, subareolar; 44 months after initial diagnosis	Multiple myeloma	–
16	Buisman et al. [129]	2016	7	–	–	Retrospective 30-year study (1985–2014); 7 male cases among 47 patients (15%)	–
17	Luo et al. [130]	2014	2	–	–	–	–
18	DeLair et al. [131]	2012	13	–	–	Retrospective 30-year study (1990–2010); males 15% of cases; prostate most common primary	–
19	Surov et al. [11]	2011	8	–	–	–	–
20	Smyrniotis et al. [132]	2005	1	–	–	–	–
21	Yeh et al. [8]	2004	2	–	–	Breast metastases associated with diffuse metastatic disease	Median survival 4 months

**Table 10:** Key immunohistochemical markers for male breast metastases by primary tumor [126,131].

Primary Tumor	Key Immunohistochemical Markers
Primary breast carcinoma	ER (+/−), PR (+/−), HER2 (+/−), GATA-3 (+), GCDFP-15 (+)
Lung adenocarcinoma	TTF-1 (+), Napsin A (+), CK7 (+), CK20 (−)
Small cell lung carcinoma	TTF-1 (+), CD56 (+)
Melanoma	HMB-45 (+), Melan-A (+), S100 (+), SOX10 (+)
Colorectal carcinoma	CDX2 (+), CK20 (+), CK7 (+/−)
B-cell lymphoma	CD20 (+), CD79a (+)
Renal cell carcinoma	PAX8(+), RCC marker (+)
Prostate adenocarcinoma	PSA (+), PSAP (+), NKX3.1 (+), AR (+)

## Data Availability

All data relevant to this systematic review are included in the article and its accompanying tables. No analytic code was generated or used during the review process.

## Supplementary Materials

The following supporting information can be downloaded at: https://www.mdpi.com/article/10.3390/cancers18152440/s1. Section S1: Table S1. PRISMA 2020 Main Checklist [133]. Table S2. PRIMSA Abstract Checklist. Section S2: Table S3. The Joanna Briggs Institute (JBI) critical appraisal tool [134].
