# Supplementary material for: Metastases to the Male Breast: A Systematic Review
Source: Cancers (Basel). 2026 Jul 29;18(15):2440. doi: 10.3390/cancers18152440 (PMC13464112; doi:10.3390/cancers18152440)
Supplement: Supplementary file 1 [file cancers-18-02440-s001.zip › cancers-4390475-supplementary.pdf]

Supplementary Materials

Supplementary Section S1

Table S1. PRISMA 2020 Main Checklist [133].

Topic	No.	Item	Location Where Item Is Reported
**TITLE**			
**Title**	1	Identify the report as a systematic review.	page 1
**ABSTRACT**			
**Abstract**	2	See the PRISMA 2020 for Abstracts checklist	
**INTRODUCTION**			
**Rationale**	3	Describe the rationale for the review in the context of existing knowledge.	page 2
**Objectives**	4	Provide an explicit statement of the objective(s) or question(s) the review addresses.	page 3
**METHODS**			
**Eligibility criteria**	5	Specify the inclusion and exclusion criteria for the review and how studies were grouped for the syntheses.	page 3
**Information sources**	6	Specify all databases, registers, websites, organisations, reference lists and other sources searched or consulted to identify studies. Specify the date when each source was last searched or consulted.	page 3
**Search strategy**	7	Present the full search strategies for all databases, registers and websites, including any filters and limits used.	page 3
**Selection process**	8	Specify the methods used to decide whether a study met the inclusion criteria of the review, including how many reviewers screened each record and each report retrieved, whether they worked independently, and if applicable, details of automation tools used in the process.	page 3
**Data collection process**	9	Specify the methods used to collect data from reports, including how many reviewers collected data from each report, whether they worked independently, any processes for obtaining or confirming data from study investigators, and if applicable, details of automation tools used in the process.	page 3
**Data items**	10a	List and define all outcomes for which data were sought. Specify whether all results that were compatible with each outcome domain in each study were sought (e.g., for all measures, time points, analyses), and if not, the methods used to decide which results to collect.	n/a
	10b	List and define all other variables for which data were sought (e.g., participant and intervention characteristics, funding sources). Describe any assumptions made about any missing or unclear information.	n/a
**Study risk of bias assessment**	11	Specify the methods used to assess risk of bias in the included studies, including details of the tool(s) used, how many reviewers assessed each study and whether they worked independently, and if applicable, details of automation tools used in the process.	page 4
**Effect measures**	12	Specify for each outcome the effect measure(s) (e.g., risk ratio, mean difference) used in the synthesis or presentation of results.	n/a
**Synthesis methods**	13a	Describe the processes used to decide which studies were eligible for each synthesis (e.g., tabulating the study intervention characteristics and comparing against the planned groups for each synthesis (item 5)).	page 3
	13b	Describe any methods required to prepare the data for presentation or synthesis, such as handling of missing summary statistics, or data conversions.	n/a
	13c	Describe any methods used to tabulate or visually display results of individual studies and syntheses.	n/a

	13d	Describe any methods used to synthesize results and provide a rationale for the choice(s). If meta-analysis was performed, describe the model(s), method(s) to identify the presence and extent of statistical heterogeneity, and software package(s) used.	n/a
	13e	Describe any methods used to explore possible causes of heterogeneity among study results (e.g., subgroup analysis, meta-regression).	n/a
	13f	Describe any sensitivity analyses conducted to assess robustness of the synthesized results.	n/a
**Reporting bias assessment**	14	Describe any methods used to assess risk of bias due to missing results in a synthesis (arising from reporting biases).	page 4
**Certainty assessment**	15	Describe any methods used to assess certainty (or confidence) in the body of evidence for an outcome.	n/a
**RESULTS**			
**Study selection**	16a	Describe the results of the search and selection process, from the number of records identified in the search to the number of studies included in the review, ideally using a flow diagram.	page 4
	16b	Cite studies that might appear to meet the inclusion criteria, but which were excluded, and explain why they were excluded.	n/a
**Study characteristics**	17	Cite each included study and present its characteristics.	page 12–28
**Risk of bias in studies**	18	Present assessments of risk of bias for each included study.	page 11
**Results of individual studies**	19	For all outcomes, present, for each study: (a) summary statistics for each group (where appropriate) and (b) an effect estimate and its precision (e.g., confidence/credible interval), ideally using structured tables or plots.	page 12–28
**Results of syntheses**	20a	For each synthesis, briefly summarise the characteristics and risk of bias among contributing studies.	page 4,5,11
	20b	Present results of all statistical syntheses conducted. If meta-analysis was done, present for each the summary estimate and its precision (e.g., confidence/credible interval) and measures of statistical heterogeneity. If comparing groups, describe the direction of the effect.	page 4,5
	20c	Present results of all investigations of possible causes of heterogeneity among study results.	n/a
	20d	Present results of all sensitivity analyses conducted to assess the robustness of the synthesized results.	n/a
**Reporting biases**	21	Present assessments of risk of bias due to missing results (arising from reporting biases) for each synthesis assessed.	page 11
**Certainty of evidence**	22	Present assessments of certainty (or confidence) in the body of evidence for each outcome assessed.	n/a
**DISCUSSION**			
**Discussion**	23a	Provide a general interpretation of the results in the context of other evidence.	page 6,7
	23b	Discuss any limitations of the evidence included in the review.	page 11
	23c	Discuss any limitations of the review processes used.	page 11
	23d	Discuss implications of the results for practice, policy, and future research.	page 10,11
**OTHER INFORMATION**			
**Registration and protocol**	24a	Provide registration information for the review, including register name and registration number, or state that the review was not registered.	page 4
	24b	Indicate where the review protocol can be accessed, or state that a protocol was not prepared.	page 4
	24c	Describe and explain any amendments to information provided at registration or in the protocol.	n/a
**Support**	25	Describe sources of financial or non-financial support for the review, and the role of the funders or sponsors in the review.	page 29
**Competing interests**	26	Declare any competing interests of review authors.	page 29
**Availability of data, code and other materials**	27	Report which of the following are publicly available and where they can be found: template data collection forms; data extracted from included studies; data used for all analyses; analytic code; any other materials used in the review.	page 29

Table S2. PRIMSA Abstract Checklist.

Topic	No.	Item	Reported?
**TITLE**			
**Title**	1	Identify the report as a systematic review.	Yes
**BACKGROUND**			
**Objectives**	2	Provide an explicit statement of the main objective(s) or question(s) the review addresses.	Yes
**METHODS**			
**Eligibility criteria**	3	Specify the inclusion and exclusion criteria for the review.	Yes
**Information sources**	4	Specify the information sources (e.g., databases, registers) used to identify studies and the date when each was last searched.	Yes
**Risk of bias**	5	Specify the methods used to assess risk of bias in the included studies.	No
**Synthesis of results**	6	Specify the methods used to present and synthesize results.	Yes
**RESULTS**			
**Included studies**	7	Give the total number of included studies and participants and summarise relevant characteristics of studies.	Yes
**Synthesis of results**	8	Present results for main outcomes, preferably indicating the number of included studies and participants for each. If meta-analysis was done, report the summary estimate and confidence/credible interval. If comparing groups, indicate the direction of the effect (i.e., which group is favoured).	Yes
**DISCUSSION**			
**Limitations of evidence**	9	Provide a brief summary of the limitations of the evidence included in the review (e.g., study risk of bias, inconsistency and imprecision).	No
**Interpretation**	10	Provide a general interpretation of the results and important implications.	Yes
**OTHER**			
**Funding**	11	Specify the primary source of funding for the review.	No
**Registration**	12	Provide the register name and registration number.	No

n/a: not applicable.

Supplementary Section S2

Table S3. The Joanna Briggs Institute (JBI) critical appraisal tool [134].

Study	Q1	Q2	Q3	Q4	Q5	Q6	Q7	Q8
Alacacioglu et al., 2012	Y	U	U	U	U	U	U	Y
Allen et al., 1991	Y	Y	Y	Y	Y	Y	Y	Y
Altintoprak et al., 2011	Y	Y	Y	Y	Y	Y	Y	Y
Bacchi et al., 2013	U	U	Y	U	U	U	U	Y
Benson et al., 1957	Y	U	Y	Y	Y	Y	Y	Y
Bruscagnin et al. 1997	U	U	U	U	U	U	U	U
Buisman et al., 2016	Y	U	U	Y	Y	U	Y	Y
Campell et al., 1962	Y	Y	Y	Y	Y	Y	Y	Y
Cangiarella et al., 1998	Y	Y	Y	Y	Y	N	Y	Y
Cappabianca et al., 2000	Y	Y	Y	Y	Y	N	U	Y
Cheng et al., 2006	Y	Y	Y	Y	Y	Y	Y	Y
Cheng et al., 2020	Y	Y	Y	Y	Y	Y	Y	Y
Chim et al., 2000	Y	Y	Y	Y	U	N	U	Y
Cooper et al., 1994	U	U	U	U	U	U	U	U
David et al., 2002	Y	Y	Y	Y	Y	Y	Y	Y
Delair et al., 2012	Y	U	Y	Y	Y	Y	Y	Y
Deeley et al., 1965	Y	U	Y	Y	Y	Y	Y	Y
Deshpande et al., 1999	Y	Y	Y	Y	Y	Y	Y	Y
Domanski, 1996	Y	Y	Y	Y	Y	U	U	Y
Drelichman et al., 1980	Y	Y	Y	Y	Y	Y	Y	Y
Erhammci et al., 2016	Y	Y	Y	Y	Y	Y	Y	Y
Franceschini et al., 2016	Y	Y	Y	Y	Y	Y	Y	Y
Fraulini et al., 2005	Y	Y	Y	Y	Y	Y	Y	Y
Fulciniti et al., 2008	Y	Y	Y	Y	Y	U	U	Y
Garza-Guajardo et al., 2005	Y	Y	Y	Y	U	N	N	Y
Gibbons et al., 1995	Y	Y	Y	Y	Y	Y	Y	Y
Gomez-Caro et al., 2006	Y	Y	Y	Y	Y	Y	Y	Y
Grigoropoulos et al., 2019	Y	Y	Y	Y	Y	Y	Y	Y
Green et al., 1991	Y	Y	Y	Y	Y	Y	Y	Y
Gur et al., 2019	Y	Y	Y	Y	Y	Y	Y	Y

Hachisuka et al., 2014	Y	Y	Y	Y	Y	Y	Y	Y
Hashmi et al., 2026	Y	Y	Y	U	U	U	U	Y
Hamby et al., 1991	Y	Y	Y	Y	Y	Y	Y	Y
Hartley et al., 1971	Y	U	U	U	U	U	U	Y
Hejmadi et al., 2003	Y	U	Y	Y	U	U	U	Y
Hesdorffer et al., 1987	U	U	U	U	U	U	U	Y
Ho et al., 2009	Y	Y	Y	Y	Y	Y	Y	Y
Hoang et al., 2025	Y	Y	Y	Y	Y	Y	Y	Y
Hsieh et al., 2005	Y	Y	Y	Y	Y	Y	Y	Y
Ishikawa et al., 2020	Y	Y	Y	Y	Y	Y	Y	Y
Kalli et al., 2016	Y	Y	U	U	U	U	U	U
Kang et al., 2014	Y	Y	Y	Y	Y	Y	Y	Y
Khazai et al., 2016	Y	Y	Y	Y	Y	Y	Y	Y
Kitano et al. 1983	Y	Y	Y	Y	Y	Y	Y	Y
Klingen et al. 2009	Y	Y	Y	Y	Y	Y	Y	Y
Koh et al. 2024	Y	Y	Y	Y	Y	Y	Y	Y
Kohi et al., 2013	Y	Y	Y	Y	Y	Y	Y	Y
Kubo et al., 2018	Y	Y	Y	Y	Y	Y	Y	Y
Kumar et al., 1986	Y	Y	Y	Y	Y	Y	U	Y
Lahham et al., 2025	Y	Y	Y	Y	Y	Y	Y	Y
Lear et al., 1980	U	U	U	U	U	U	U	U
Lee et al., 2007	Y	Y	Y	U	U	N	N	Y
Lee et al., 2011	Y	U	Y	Y	Y	Y	Y	Y
Lin et al., 2016	Y	Y	Y	Y	Y	Y	Y	Y
Lo et al., 2004	Y	Y	Y	U	U	Y	Y	Y
Lome et al., 1970	U	U	U	U	U	U	U	Y
Luo et al., 2014	U	U	U	U	U	U	U	U
Lopez-Guerra et al., 2008	Y	Y	Y	Y	Y	Y	Y	Y
Mokoala et al., 2019	Y	U	N	U	Y	Y	N	N
Moldwin et al., 1989	Y	Y	Y	Y	Y	Y	Y	Y
Monaco et al., 2009	Y	Y	Y	Y	Y	Y	Y	Y
Moreno-Astudillo et al., 2019	Y	Y	Y	Y	Y	Y	Y	Y
Mun et al., 2014	Y	Y	Y	Y	U	U	U	Y
Muttarak et al., 1998	Y	Y	Y	Y	Y	Y	Y	Y

Nair et al., 2005	Y	Y	Y	Y	N	N	N	Y
Narese et al., 2014	Y	Y	Y	U	U	U	U	Y
Naritoku et al., 1983	Y	Y	Y	Y	Y	Y	Y	Y
Njiaju et al., 2010	Y	Y	Y	Y	Y	Y	Y	Y
Portaluri et al., 2022	Y	Y	Y	Y	Y	Y	Y	Y
Pribe et al., 1963	Y	Y	Y	Y	Y	Y	Y	Y
Prieto et al. 2005	Y	Y	Y	Y	U	U	U	Y
Ramamurthy et al., 1991	Y	Y	Y	Y	Y	Y	Y	Y
Ramar et al., 2001	Y	Y	Y	Y	Y	Y	Y	Y
Roy et al. 2018	Y	Y	Y	Y	Y	Y	Y	Y
Sahoo et al., 2001	Y	Y	Y	Y	Y	Y	Y	Y
Salyer et al., 1973	Y	Y	Y	Y	Y	Y	Y	Y
Santana Valenciano et al., 2023	Y	Y	Y	Y	Y	U	Y	Y
Scoot et al., 1974	Y	Y	Y	Y	Y	Y	Y	Y
Seipel et al., 1972	Y	Y	Y	Y	Y	Y	Y	Y
Siddiqui et al., 2002	U	U	Y	U	U	U	U	Y
Shah et al., 2014	U	U	Y	Y	Y	Y	Y	Y
Shukla et al., 2005	Y	Y	Y	N	N	N	N	Y
Sippo et al., 2016	Y	Y	Y	N	U	U	N	Y
Smyrniotis et al., 2005	Y	Y	Y	Y	U	U	U	Y
Sneige et al., 1989	Y	Y	Y	Y	Y	Y	Y	Y
Sun et al., 2018	Y	U	Y	Y	Y	Y	Y	Y
Surov et al., 2011	Y	Y	Y	Y	Y	Y	Y	Y
Toombs et al., 1977	Y	U	Y	Y	Y	Y	Y	Y
Tsung, 2017	Y	U	Y	Y	Y	N	N	Y
Ucar et al., 2007	Y	Y	U	U	U	U	U	Y
Verger et al., 1992	Y	Y	Y	Y	Y	U	N	Y
Wan et al., 2020	Y	Y	Y	Y	Y	Y	Y	Y
Wood et al., 2008	Y	Y	Y	Y	Y	Y	Y	Y
Wang T. et al., 2011	Y	Y	Y	Y	Y	Y	Y	Y
Yan et al., 2000	Y	Y	Y	Y	Y	Y	Y	Y
Yeh et al., 2004	U	U	U	U	U	U	U	Y
Yoon et al., 2021	Y	Y	Y	Y	Y	Y	Y	Y
Zhang et al., 2025	Y	Y	Y	Y	Y	Y	Y	Y

Case Reports Critical Appraisal Tool Answers: Yes, No, Unclear or Not/Applicable.

Questions:

1. Were patient's demographic characteristics clearly described?
2. Was the patient's history clearly described and presented as a timeline?
3. Was the current clinical condition of the patient on presentation clearly described?
4. Were diagnostic tests or methods and the results clearly described?
5. Was the intervention(s) or treatment procedure(s) clearly described?
6. Was the post-intervention clinical condition clearly described?
7. Were adverse events (harms) or unanticipated events identified and described?
8. Does the case report provide takeaway lessons?
